# Supplementary material for: Extensive sampling reveals the phenology and habitat use of Afrotropical parasitoid wasps (Hymenoptera: Ichneumonidae: Rhyssinae)
Source: R Soc Open Sci. 2019 Aug 14;6(8):190913. doi: 10.1098/rsos.190913 (PMC6731719; doi:10.1098/rsos.190913)
Supplement: Figures S1–S6 from "Extensive sampling reveals the phenology and habitat use of Afrotropical parasitoid wasps (Hymenoptera: Ichneumonidae: Rhyssinae)" [file rsos190913supp1.pdf]

S1

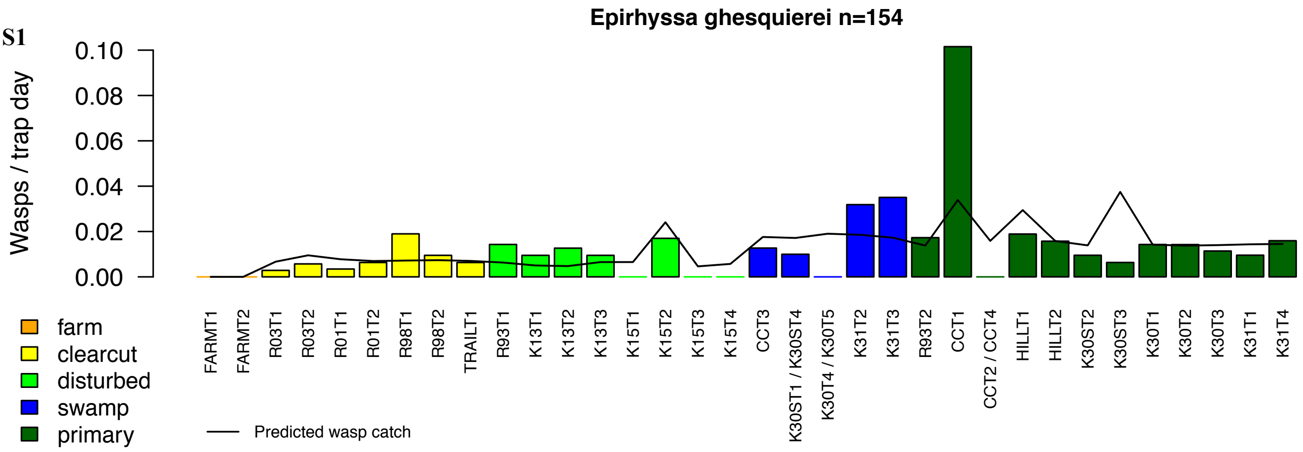
Epirhyssa ghesquierei n=154
Wasps / trap day
0.10
0.08
0.06
0.04
0.02
0.00
farm
clearcut
disturbed
swamp
primary
Predicted wasp catch
FARMT1
FARMT2
R03T1
R03T2
R01T1
R01T2
R98T1
R98T2
TRAILT1
R93T1
K13T1
K13T2
K13T3
K15T1
K15T2
K15T3
K15T4
CCT3
K30ST1 / K30ST4
K30T4 / K30T5
K31T2
K31T3
R93T2
CCT1
CCT2 / CCT4
HILLT1
HILLT2
K30ST2
K30ST3
K30T1
K30T2
K30T3
K31T1
K31T4

S2

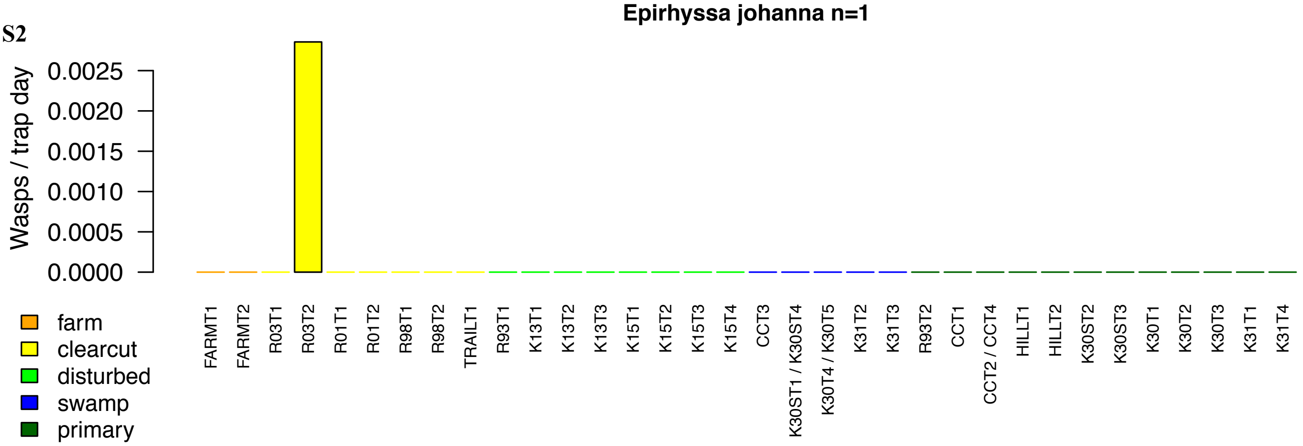
Epirhyssa johanna n=1
Wasps / trap day
0.0025
0.0020
0.0015
0.0010
0.0005
0.0000
farm
clearcut
disturbed
swamp
primary
FARMT1
FARMT2
R03T1
R03T2
R01T1
R01T2
R98T1
R98T2
TRAILT1
R93T1
K13T1
K13T2
K13T3
K15T1
K15T2
K15T3
K15T4
CCT3
K30ST1 / K30ST4
K30T4 / K30T5
K31T2
K31T3
R93T2
CCT1
CCT2 / CCT4
HILLT1
HILLT2
K30ST2
K30ST3
K30T1
K30T2
K30T3
K31T1
K31T4

S3

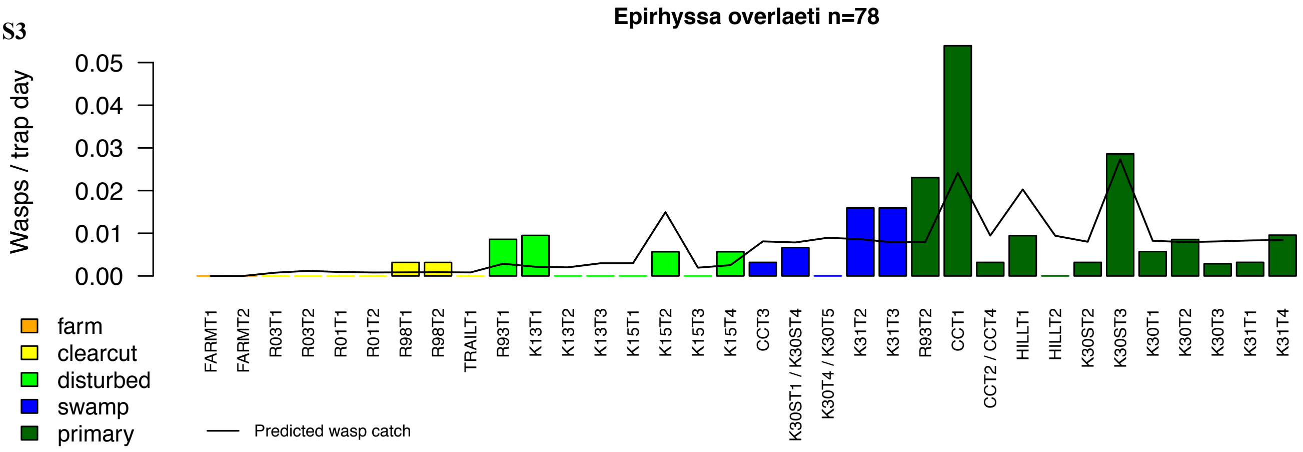
Epirhyssa overlaeti n=78
Wasps / trap day
0.05
0.04
0.03
0.02
0.01
0.00
farm
clearcut
disturbed
swamp
primary
Predicted wasp catch
FARMT1
FARMT2
R03T1
R03T2
R01T1
R01T2
R98T1
R98T2
TRAILT1
R93T1
K13T1
K13T2
K13T3
K15T1
K15T2
K15T3
K15T4
CCT3
K30ST1 / K30ST4
K30T4 / K30T5
K31T2
K31T3
R93T2
CCT1
CCT2 / CCT4
HILLT1
HILLT2
K30ST2
K30ST3
K30T1
K30T2
K30T3
K31T1
K31T4

S4

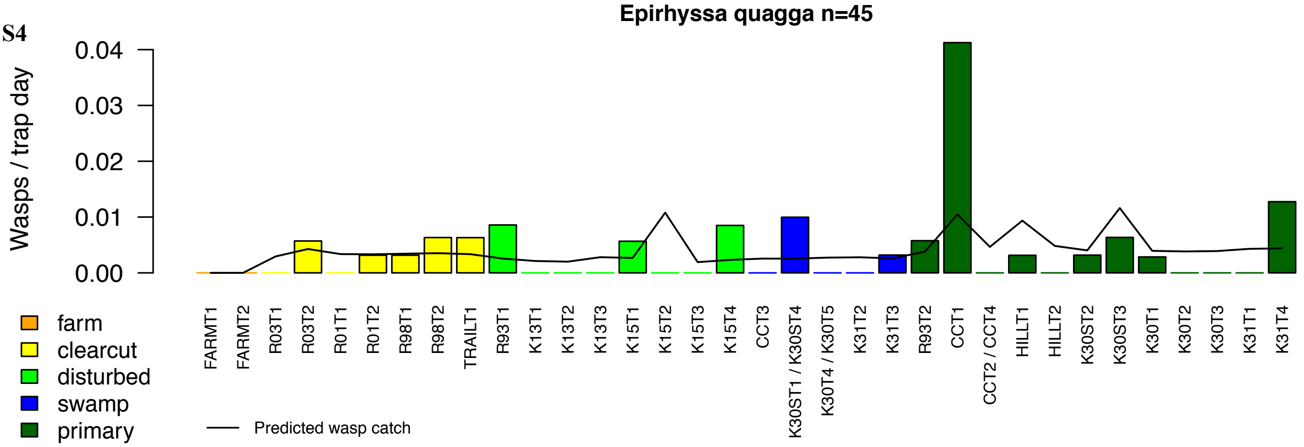

S5

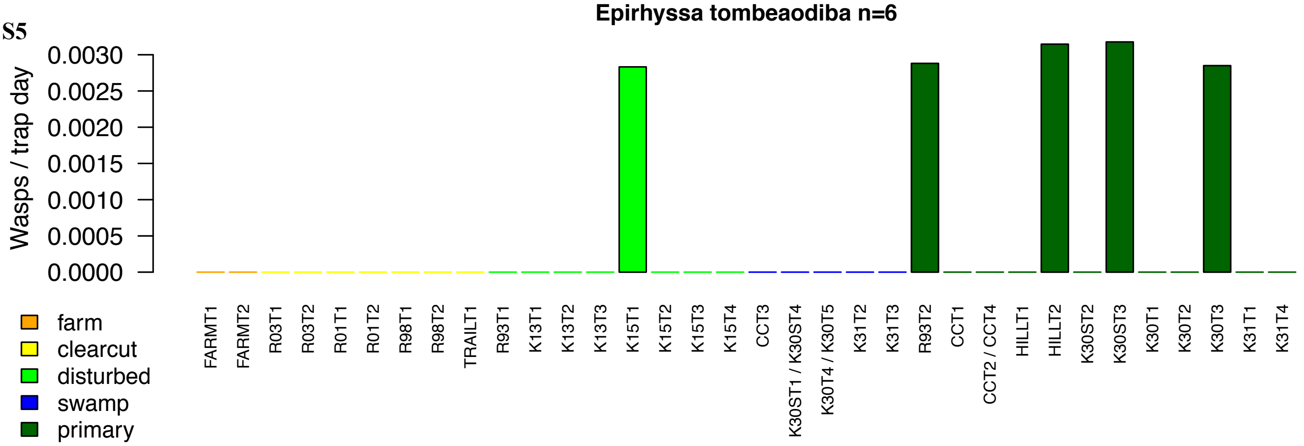

S6

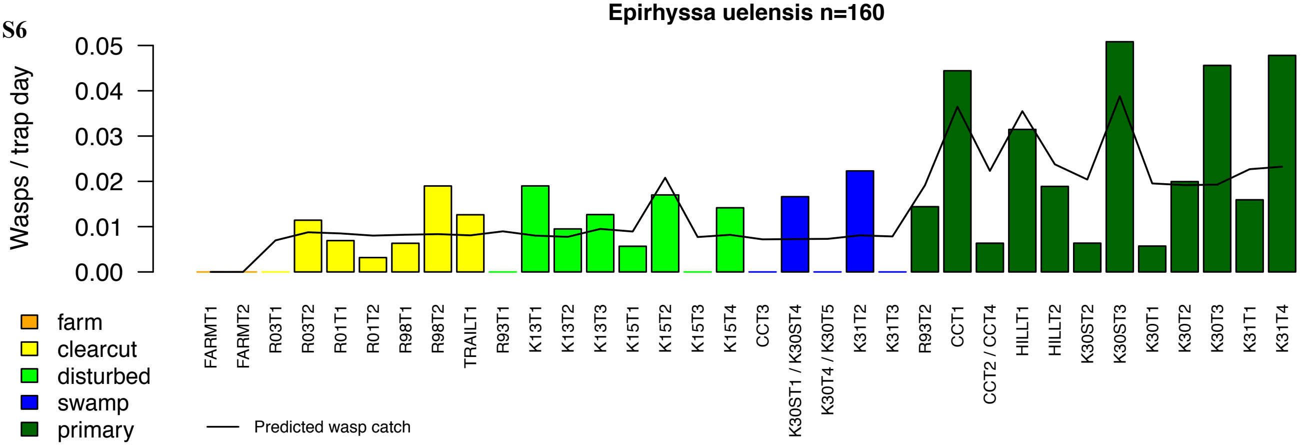

Figures S1–6. Number of rhyssines of each species caught in 34 traps (as individuals per trap per day). Catches were generally highest in the primary forest trap CCT1 and lowest in farmland. The black line shows the catch predicted by a model taking into account the date, rainfall, amount of decaying wood and forest type. The predicted catches are not shown for two species due to too low sample size. Catches after 4 Sep 2015 were not included in the model (due to lack of weather data) and are not shown.
